# A simple immunohistochemical panel comprising 2 conventional markers, Ki67 and p53, is a powerful tool for predicting patient outcome in luminal-type breast cancer

**DOI:** 10.1186/1472-6890-13-5

**Published:** 2013-02-06

**Authors:** Takayuki Kobayashi, Keiichi Iwaya, Tomoyuki Moriya, Tamio Yamasaki, Hitoshi Tsuda, Junji Yamamoto, Osamu Matsubara

**Affiliations:** 1 Department of Basic Pathology, National Defense Medical College, 3-2 Namiki, Tokorozawa, Saitama, 359-8513, Japan; 2 Department of Surgery, National Defense Medical College, 3-2 Namiki, Tokorozawa, Saitama, 359-8513, Japan; 3 Pathology and Clinical Laboratory Division, National Cancer Center Hospital, 5-1-1 Tsukiji, Chuo-ku, Tokyo, 104-0045, Japan

**Keywords:** Ki67 labeling index, p53, IHC panel, Luminal-type breast cancer

## Abstract

**Background:**

Ki67 is widely used in order to distinguish the “A” and “B” subtypes of luminal-type breast cancer. This study aimed to validate the prognostic value of adding p53 to Ki67 for characterizing luminal-type breast cancer.

**Methods:**

Immunostaining for Ki67, p53, and the molecular markers HER2, CK5/6, CK14, EGFR, FOXA1, GATA3, and P-cadherin was examined hormone receptor (HR)-positive cancer tissues from 150 patients. The prognostic value of an immunohistochemical panel comprising Ki67 and p53 was compared with that of the single Ki67 labeling index (LI), and uni- and multivariate analyses were performed.

**Results:**

Division of the patients based on the immunohistochemistry results into favorable- (low Ki67 LI, p53-negative) and unfavorable- (high Ki67 LI and/or p53-positive) phenotype groups yielded distinctly different Kaplan-Meier's curves of both disease-free (*P*<0.0001) and overall survival (*P*=0.0007). These differences were much more distinct than those between the corresponding low Ki67 LI vs. high Ki67LI curves. While the prognostic values of the other molecular markers were not significant, combined Ki67-p53 status was an independent prognostic factor by multivariate analysis.

**Conclusion:**

These data indicate that an immunohistochemical panel comprising Ki67 and p53 is a practical tool for management of patients with HR-positive breast cancer.

## Background

Approximately 70% of breast cancers express a hormone receptor (HR). HR status is a powerful predictor of response to therapies that inhibit estrogen synthesis or block the action of its receptor [1]. Endocrine therapies are established in the adjuvant setting [2-4]. For example, women with node-negative, HR-positive breast cancer who are treated with tamoxifen alone after surgery have an average 10-year recurrence rate of only 15% [5]. If all of these patients were offered chemotherapy, 85% would be over-treated [6]. It is therefore important to distinguish patients with HR-positive tumors at high risk for recurrence who need additional chemotherapy from those for whom adjuvant hormonal therapy alone may suffice [7].

Multi-gene assays are strong candidate tools for predicting the risk of recurrence in HR-positive patients. For example, the Oncotype DX™ assay analyzes the expression levels of 21 genes (including 5 reference genes) in formalin-fixed paraffin embedded tissues and produces a Recurrence Score (RS) that predicts the likelihood of distant recurrence [6] and the benefit of chemotherapy in women with early HR-positive breast cancer [8]. Although Oncotype DX™ is a potentially powerful tool for stratification of HR-positive patients, it is too expensive to use in routine clinical practice. Many oncologists are eager for an alternative assay that is inexpensive as well as easy to use; one possible approach would be an immunohistochemical (IHC) assay.

Sorlie et al. reported that breast cancers could be divided, based on their gene expression profiles, into at least 4 groups: luminal-type, HER2-type, normal-like-type, and basal-type. Luminal-type cancers are characterized by an activated estrogen receptor (ER) signaling pathway and are divided into 2 subtypes, luminal subtypes “A” and “B. In general, luminal-subtype-A tumors express higher levels of ER and carry a better prognosis than do luminal-subtype-B tumors [9]. Recent studies have shown that tumors of luminal subtype A have a lower rate of *p53* mutation [9-12] and are less proliferative [13,14] than those of luminal subtype B, suggesting that the combination of p53 status and proliferation markers could be useful to distinguish between luminal subtypes A and B.

The tumor suppressor gene *p53* plays a most important role in regulating normal cell fate in response to various stresses, and disruption of p53 function is often involved in tumor progression. Since the co-authors first reported in 1991 that distinct immunoreaction with p53 in the nuclei of breast cancer cells is an independent prognostic indicator [15], more than 1000 articles about the correlation between p53 status and breast cancer prognosis have been published. Recent studies have shown that abnormalities of the *p53* gene [16] and accumulation of p53 protein in the nuclei [17,18] are also robust prognostic indicators in HR-positive patients.

Ki67 is the marker most often used to evaluate tumor proliferation status. The Ki67 protein is a large (395 kD) nuclear protein that is present during all active phases of the cell cycle except for the G0 phase. Because proliferation status is closely correlated with tumor aggressiveness, the Ki67 labeling index (LI) is considered an established prognostic marker for various tumor types, including breast cancer [19,20]. Previous clinical studies have revealed Ki67 LI to be a good prognostic indicator for HR-positive breast cancer patients [7,21].

Although Ki67 is a strong prognostic indicator for HR-positive breast cancer patients, adding Ki67 to the commonly used indices in daily practice is controversial [19]. Furthermore, its predictive value is weaker than that of multi-gene expression assays such as Oncotype DX™. In this study, we attempted to validate the classification of HR-positive breast cancer patients by combined analysis of Ki67 LI and p53 status. We performed immunohistochemical examination of Ki67 and p53 expression in 150 samples of surgically resected HR-positive invasive breast cancers and analyzed the relationships between combined Ki67-p53 status and clinicopathological factors, including prognosis.

## Methods

### Patients and Samples

Of the 247 patients who had undergone mastectomy or breast-conserving surgery for invasive ductal carcinoma of the breast at the National Defense Medical College (NDMC) Hospital between 1995 and 1999, 150 patients with ER-positive and/or progesterone-receptor (PgR)-positive localized breast carcinomas were selected based on immunohistochemical reevaluation of ER and PgR expression. Tissue microarray (TMA) blocks of the tumors from these 150 patients were constructed as previously described [22]. Briefly, double tissue cores 2 mm in diameter were taken from each donor block, and these core specimens were transferred to a recipient block using a Tissue Microarrayer (Beecher Instruments, Silver Spring, MD, USA). The use of the tissue blocks was internally reviewed and approved by the NDMC Ethics Committee.

The 150 patients had been followed up for a median of 82 months (range, 1–151 months), during which time there were 30 relapses and 15 deaths. In most cases, the patients were prescribed adjuvant endocrine therapy (for example, tamoxifen, toremifene, fadrozole, or LHRH analogues). Forty-nine patients with large tumors and/or 4 or more lymph node metastases had received adjuvant chemotherapy (cyclophosphamide-epirubicin-5-fluorouracil (CEF), cyclophosphamide-adriamycin-5-fluorouracil (CAF), cyclophosphamide-methotrexate-5-fluorouracil (CMF), or oral fluoropyrimidines), and 12 patients with locally advanced breast cancer had received preoperative chemotherapy (for example, CAF or CEF). One hundred forty-eight patients were females and 2 were males. The clinical stage of the patients was determined based on the TNM classification according to general rules of the Japanese Breast Cancer Society [23]. Clinicopathological data were obtained from the medical records and pathology reports, but ER, PgR and HER2 status were examined in our previous study [22].

### Immunohistochemistry

Immunohistochemistry was performed on a TMA composed of 150 breast cancer tissue specimens. The antibodies used were mouse monoclonal anti-p53 antibody (DO-7, Dako, Glostrup, Denmark), mouse monoclonal anti-Ki67 antibody (MIB-1, Dako), mouse monoclonal anti-FOXA1 antibody (2D7, Abnova, Taipei, Taiwan), mouse monoclonal anti-GATA3 antibody (HG3-31, Santa Cruz, Santa Cruz, CA, USA) mouse monoclonal anti-CK5/6 antibody (D5/16 B4, Dako), mouse monoclonal anti-CK14 antibody (LL002, NeoMarkers, Fremont, CA, USA), mouse monoclonal anti-P-cadherin antibody (56C1, Novocastra, Newcastle, UK), and a mouse monoclonal anti-EGFR antibody included in an EGFR pharmDX kit (Dako).

Sections (4-um-thick) were cut from the formalin-fixed, paraffin-embedded TMA blocks. Antigens were retrieved by microwave heating for 30 min in 10 mM sodium citrate (pH 6.0) for CK5/6 and GATA3 or by autoclaving for 20 min in 10 mM Tris–HCl (pH 9.0) for Ki67, p53, CK14, FOXA1, and P-cadherin. To block endogenous peroxidase activity, the sections were treated for 5 min with 100% methanol containing 3% H_2_O_2_. Non-specific binding was blocked by incubation in 1% normal swine serum (Dako) in phosphate-buffered saline. The slides were incubated with primary antibodies at 4°C overnight and then reacted with a dextran polymer reagent combined with secondary antibodies and peroxidase (Envision Plus; Dako) for 30 min at room temperature. Specific antigen-antibody reactions were visualized with 0.2% diaminobenzidine tetrahydrochloride and hydrogen peroxide. Immunostaining for EGFR was performed in accordance with the package inserts of the EGFR pharmDX Kit. The sections were counterstained with Mayer’s hematoxylin.

### Evaluation of immunohistochemistry

Although there is no universal cut-off value for Ki67 LI, Cheang et al. showed that, using the cases which were subtyped by gene expression profile, the best Ki67 LI cut-off value to distinguish luminal B from luminal A was 13% [7]. Furthermore, similar to the 10% cut-off value was used in several reports [21,24-28]. So, in this study, Ki67 LI greater than 10% was classified as high. The Ki67 LI was calculated as the percentage of positive tumor nuclei divided by the total number of tumor cells examined on the basis of a manual count of 500 or more cells under high power (400×).

For p53, FOXA1, and GATA3, cells with immunostaining in the nucleus were defined as positive, while for CK5/6, CK14, and P-cadherin, cells with immunostaining along the cellular periphery and/or in the cytoplasm were defined as positive. For p53, positive staining of fewer than 10% of the tumor cells was defined as negative tumor expression and staining of 10% or more of the tumor cells as positive tumor expression [15]. For P-cadherin, membrane staining of fewer than 50% of the tumor cells was defined as negative tumor expression and staining of 50% or more of the tumor cells as positive tumor expression. P-cadherin positive tumors were further divided into “weakly” and “strongly” expressing tumors based on staining intensity. Finally, negative and weakly P-cadherin-staining tumors were classified as “low” and strongly P-cadherin-staining tumors as “high”. A tumor expression cutoff point of 10% of cells stained was used for GATA3, CK5/6, and CK14 and a cutoff of 70% of cells stained for FOXA1, regardless of staining intensity. An EGFR score of 0–3+ was assigned according to the manufacturer’s package insert, and scores of 1–3+ were classified as positive. CK5/6, CK14, and EGFR were considered basal phenotype markers.

ER and PgR were examined immunohistochemically as described in the previous study [22], using mouse monoclonal anti-human ER (clone 1D5, Dako) and mouse anti-human PgR (clone PgR636, DAKO). ER and PgR were defined as positive if the nuclear staining was seen in 10% or more of tumor cells. Hormone receptor positive was defined as at least one of ER or PgR positive, and hormone receptor negative was defined as ER and PgR negative. HER2 was evaluated by IHC using rabbit polyclonal anti-HER2 antibody (HercepTest kit, Dako) and FISH (in case of IHC 2+) using Path Vysion kit (Abbott Park, IL, USA). HER2 was defined as positive if the IHC score was “3+” according to the standard procedure, or gene amplification (HER2:CEP17 ratio > 2.0) was detected by FISH [29].

The immunohistochemistry results were evaluated independently by 2 observers (TK and KI), and cases with disparate scores were re-evaluated and discussed until a consensus was reached. Ki67-positive cells were counted and the labeling index calculated by TK alone.

### Statistical analysis

Comparisons between groups were evaluated with the chi-squared test or Fisher’s exact test. Patient survival curves were drawn using the Kaplan-Meier method and analyzed by the log-rank test. The hazard ratios and corresponding 95% confidence intervals (CIs) were calculated with Cox’s proportional hazards model. Univariate and multivariate Cox’s proportional hazards models were used to explore the associations of variables with disease-free and overall survival. For all tests, differences at *P* < 0.05 were considered statistically significant. All analyses were performed using the software JMP 6.0 for Windows (SAS Institute Inc., Cary, NC, USA).

## Results

### Expression of markers (Ki67, p53, HER2, FOXA1, GATA3, CK5/6, CK14, EGFR, and P-cadherin) in HR-positive tumors (n=150)

**Figure 1:**
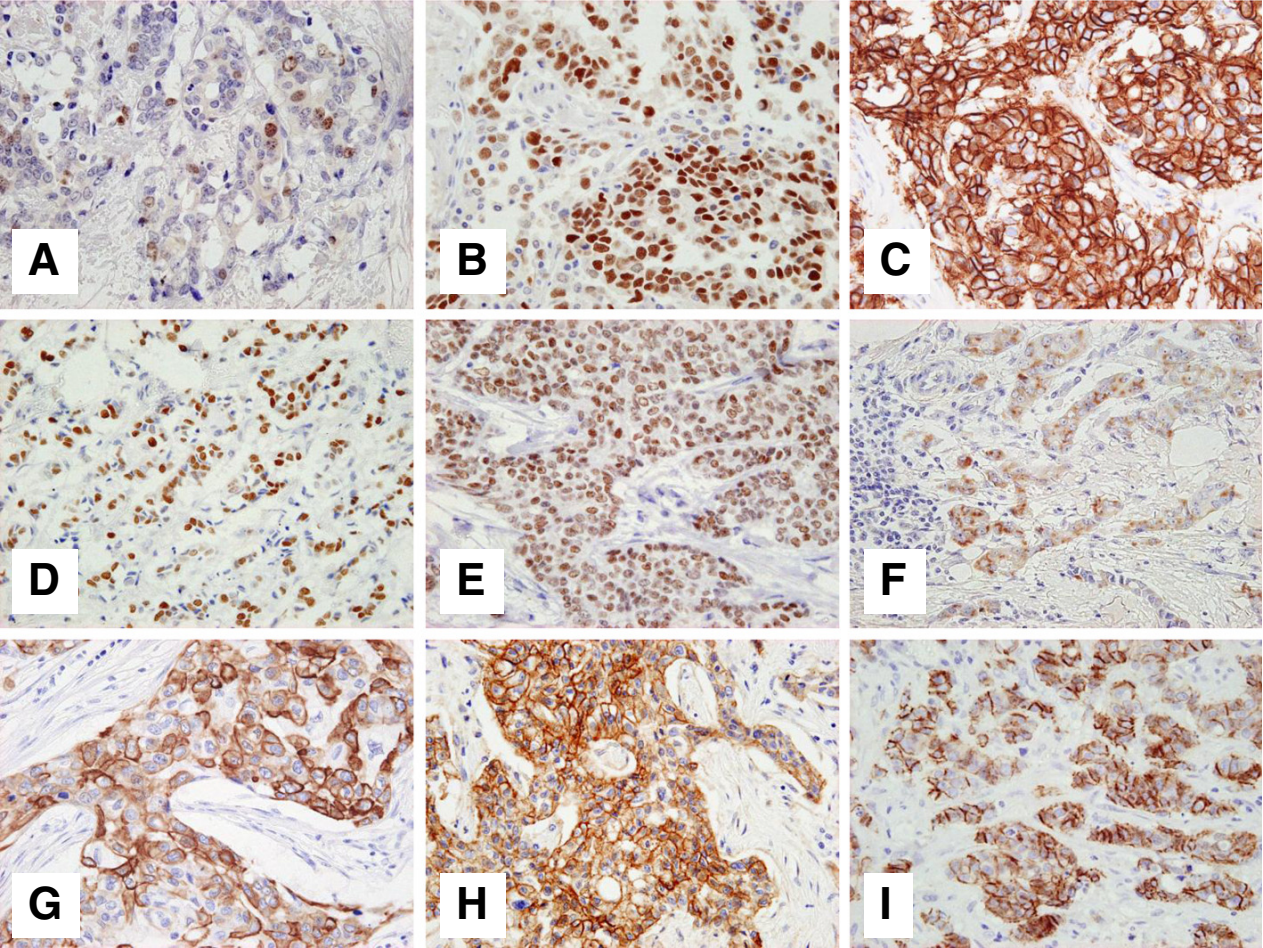
**Representative images of immunostaining for 9 molecular markers.A**, Positive nuclear Ki67 staining. The Ki67 labeling index of this specimen is 12%. **B**, p53 staining in the nucleus. This tumor was scored as p53-positive. **C**, Positive HER2 membrane staining. The HER2 expression of this tumor was scored as 3+. **D**, Positive nuclear FOXA1 staining. This tumor was classified as FOXA1-positive. **E**, Positive nuclear GATA3 staining. This tumor was classified as GATA3-positive. **F**, Positive CK5/6 membrane or sub-membrane staining of tumor cells. This tumor was classified as CK5/6-positive. **G**, Positive CK14 membrane or sub-membrane staining of tumor cells. This tumor was classified as CK14-positive. **H**, Positive EGFR membrane staining of tumor cells. The EGFR expression of this tumor was scored as 3+. **I**, Positive P-cadherin membrane or sub-membrane immunoreactivity of tumor cells. This tumor was classified as P-cadherin-high. The magnification of all figures is ×400.

Representative images of immunostaining for the markers examined in this study are shown in Figure 1. Among 150 HR-positive tumors, there were 51 (34%) Ki67 LI-high tumors, 22 (15%) p53-positive tumors, 127 (85%) FOXA1-positive tumors, 120 (80%) GATA3-positive tumors, and 6 (4%) CK5/6-positive, 3 (2%) CK14-positive, 3 (2%) EGFR-positive, and 56 (37%) P-cadherin-high tumors. Ten (7%) tumors showed positive staining for at least 1 of the basal phenotype markers CK5/6, CK14, and EGFR. Eight tumors were determined as HER2-positive, which were composed of 6 tumors with IHC 3+ and 2 tumors with IHC 2+ and FISH +.

### Correlations of clinicopathological factors (tumor size, lymph-node status, nuclear grade, and molecular markers) with Ki67 LI status and p53 immunoreactivity in HR-positive tumors (n=150)

The tumors with high Ki67 LIs showed significantly higher frequencies of high nuclear grade, HER2 positivity, basal phenotype marker positivity, and P-cadherin (*P* = 0.013, *P* = 0.0010, and *P* = 0.0015, and *P* = 0.013, respectively). The tumors with positive p53 staining showed significantly higher frequencies of large tumor size and high nuclear grade (*P* = 0.0013 and *P* = 0.035, respectively).

### Correlation between clinicopathological factors and combined Ki67-p53 status in HR-positive tumors (n=150)

There were 88 (59%), 11 (7%), 40 (27%), and 11 (7%) tumors with the Ki67 LI-low and p53-negative, Ki67 LI-low and p53-positive, Ki67 LI-high and p53-negative, and Ki67 LI-high and p53-positive phenotypes, respectively. The tumors with the “favorable” Ki67 LI-low and p53-negative phenotype (n = 88) showed lower frequencies of high nuclear grade, HER2 positivity, basal phenotype marker positivity, and high P-cadherin expression (*P* = 0.0008, *P* = 0.0006, *P* = 0.0016 and *P* = 0.0019, respectively; Table 1) than did those with “unfavorable” Ki67 LI-low and p53-positive (n = 11), Ki67 LI-high and p53-negative (n = 40), and Ki67 LI-high and p53-positive (n = 11) phenotypes (n = 62 total). Interestingly, all HER2-positive tumors were shown to be unfavorable phenotype tumors. This study found no correlations between the combined Ki67-p53 status and the clinical factors tumor size, nodal status.

**Table 1:** Clinicopathological implication of Ki67-p53 combination status in surgically resected hormone receptor-positive breast cancers

**Variables**	**Cases**			
	**Ki67-p53 combination status**			
	**Total**	**Low Ki67 LIand Negative p53**	**High Ki67 LI and/or Positive p53**	***P *- value**
	**(n=150)**	**(n=88)**	**(n=62)**	
Age				
≦50	71	40	31	
>50	79	48	31	0.58
Tumor size				
<5.0 cm	128	78	50	
≧5.0 cm	19	8	11	0.12
Unknown	3	2	1	
Lymph node metastasis				
(−)	84	52	32	
(+)	63	34	29	0.33
Unknown	3	2	1	
Stage				
I or II	129	78	51	
III	17	7	10	0.13
Unknown	4	3	1	
Nuclear grade				
1, 2	115	76	39	
3	35	12	23	0.0008
HER2 status				
Negative	142	88	54	
Positive	8	0	8	0.0006
Basal phenotype marker (CK5/6, CK14, EGFR)				
Negative	140	87	53	
Positive	10	1	9	0.0016
FOXA1				
Negative	20	14	6	
Positive	127	72	55	0.38
NE	3	2	1	
GATA3				
Negative	30	18	12	
Positive	120	70	50	0.99
P-cadherin				
Low	94	62	32	
High	56	26	30	0.0019
Chemotherapy				
No	98	59	39	0.59
Yes	52	29	23	

Abbreviation: *LI* labeling index, *NE* not evaluable.

### Prognostic implications of combined Ki67-p53 status in HR-positive and HER2-negative tumors (n=142)

The patients with HER2-positive tumors could be received anti-HER2 treatments which have tremendous effect in both adjuvant and metastatic setting [30,31]. So, we next conducted the further analyses using the cases with HR-positive and HER2-negative tumors in order to evaluate more definitely the clinical implication of Ki67 and p53.

**Figure 2:**
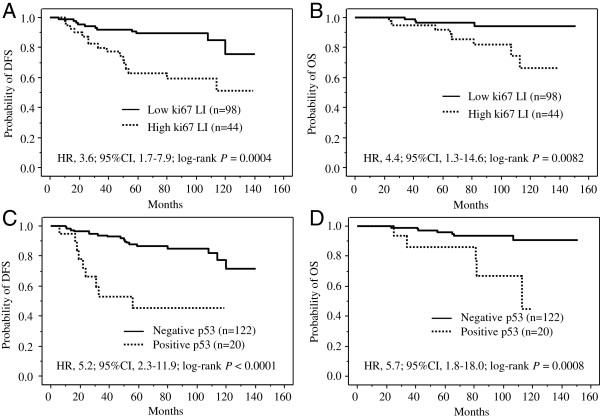
**Prognostic impact of Ki67 labeling index (LI) and p53 status in patients with hormone receptor-positive and HER2-negative breast cancer (n=142).A,** Disease free survival curves for 98 patients with Ki67 LI-low tumors and 44 patients with Ki67 LI-high tumors. The 2 curves differ significantly (HR, 3.6; 95% CI, 1.7–7.9; log-rank *P* = 0.0004). **B,** Overall survival curves for 98 patients with Ki67 LI-low tumors and 44 patients with Ki67 LI-high tumors. The 2 curves differ significantly (HR, 4.4; 95% CI, 1.3–14.6; log-rank *P* = 0.0082). **C**, Disease-free survival curves for 122 patients with p53-negative tumors and 20 patients with p53-positive tumors. The 2 curves differ significantly (HR, 5.2; 95% CI, 2.3–11.9; log-rank *P* < 0.0001). **D,** Overall survival curves for 122 patients with p53-negative tumors and 20 patients with p53-positive tumors. The 2 curves differ significantly (HR, 5.7; 95% CI, 1.8–18.0; log-rank *P* = 0.0008).

Both the disease-free survival (DFS) and overall survival (OS) curves differed significantly between the patients with Ki67 LI-low tumors and those with Ki67-LI-high tumors (DFS: HR, 3.6; 95% CI, 1.7–7.9; log-rank *P* = 0.0004; Figure 2A, and OS: HR, 4.4; 95% CI, 1.3–14.6; log-rank *P* = 0.0082; Figure 2B). Both curves also differed significantly between the patients with p53-negative tumors and those with p53-positive tumors (DFS: HR, 5.2; 95% CI, 2.3–11.9; log-rank *P* < 0.0001; Figure 2C, and OS: HR, 5.7; 95% CI, 1.8–18.0; log-rank *P* = 0.0008; Figure 2D). Furthermore, a multivariate analysis using Cox’s proportional hazard model and including immunostaining for the markers Ki67, p53, HER2, FOXA1, GATA3, the basal phenotype markers, and P-cadherin selected Ki67 and p53 as significant prognostic factors for DFS (*P* = 0.0073 and *P* = 0.0025, respectively; Table 2) and p53 for OS (*P* = 0.030, Table 2).

**Table 2:** Univariate and multivariate analyses of immunohistochemical parameters (disease-free survival and overall survival)

			**Univariate**			**Multivariate**		
		**Total (n=142)**	**Hazard ratio**	**(95% CI)**	***P*-value**	**Hazard ratio**	**(95% CI)**	***P*-value**
Disease-free survival								
Ki67 LI	Low	98	1			1		
	High	44	3.6	1.7-7.9	0.0010	3.2	1.4-7.6	0.0073
p53	Negative	122	1			1		
	Positive	20	5.2	2.3-11.9	<0.0001	3.9	1.6-9.4	0.0025
FOXA1	Low	20	1			1		
	High	119	1.4	0.51-3.6	0.54	1.7	0.56-5.2	0.34
GATA3	Negative	30	1			1		
	Positive	112	1.5	0.65-3.5	0.34	1.2	0.46-3.4	0.66
Basal phenotype marker	Negative	133	1			1		
(CK5/6, CK14, EGFR)	Positive	9	1.0	0.24-4.3	0.98	0.47	0.10-2.2	0.34
P-cadherin	Low	90	1			1		
	High	52	1.3	0.59-2.8	0.52	0.87	0.37-2.1	0.75
Overall survival								
Ki67 LI	Low	98	1			1		
	High	44	4.4	1.3-14.6	0.016	3.2	0.91-11.9	0.070
p53	Negative	122	1			1		
	Positive	20	5.7	1.8-18.0	0.0029	3.8	1.1-13.0	0.030
FOXA1	Low	20	1			1		
	High	119	0.49	0.06-3.9	0.50	0.75	0.07-7.5	0.81
GATA3	Negative	30	1			1		
	Positive	112	1.1	0.31-4.3	0.84	1.3	0.29-6.1	0.70
Basal phenotype marker	Negative	133	1			1		
(CK5/6, CK14, EGFR)	Positive	9	1.2	0.15-9.3	0.86	0.60	0.07-5.4	0.64
P-cadherin	Low	90	1			1		
	High	52	1.8	0.57-5.5	0.32	1.2	0.36-4.2	0.73

Abbreviation: *95% CI* 95% confidence interval, *LI* labeling index.

**Figure 3:**
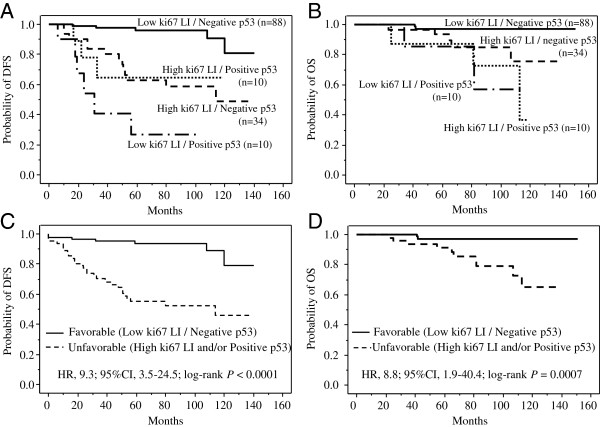
**Prognostic impact of combined Ki67-p53 status in patients with hormone receptor-positive and HER2-negative breast cancer (n=142)**. **A,** Disease-free survival curves for 88 patients with Ki67 LI-low and p53-negative tumors, 34 patients with Ki67 LI-high and p53-negative tumors, 10 patients with Ki67 LI-low and p53-positive tumors, and 10 patients with Ki67 LI-high and p53-positive tumors. Patients with Ki67 LI-low and p53-negative tumors had significantly longer disease-free survival than those with Ki67 LI-low and p53-positive tumors, those with Ki67 LI-high and p53-negative tumors, or those with Ki67 LI-high and p53-positive tumors (*P* < 0.0001, *P* < 0.0001, and *P* = 0.0005, respectively). **B,** Overall survival curves for 88 patients with Ki67 LI-low and p53-negative tumors, 34 patients with Ki67 LI-high and p53-negative tumors, 10 patients with Ki67 LI-low and p53-positive tumors, and 10 patients with Ki67 LI-high and p53-positive tumors. Patients with Ki67 LI-low and p53-negative tumors had significantly longer disease-free survival than those with Ki67 LI-low and p53-positive tumors, those with Ki67 LI-high and p53-negative tumors, or those with Ki67 LI-high and p53-positive tumors (*P* = 0.0010, *P* = 0.011, and *P* < 0.0001, respectively). **C,** Disease-free survival curves for patients with favorable-phenotype tumors (88 patients with Ki67 LI-low and p53-negative tumors) and unfavorable-phenotype tumors (54 patients with Ki67 LI-high and/or p53-positive tumors). The disease-free survival time was significantly longer in the favorable-phenotype group than in the unfavorable-phenotype group (HR, 9.3; 95% CI, 3.5–24.5; *P* < 0.0001). **D,** Overall survival curves for patients with favorable-phenotype tumors (88 patients with Ki67 LI-low and p53-negative tumors) and those with unfavorable-phenotype tumors (54 patients with Ki67 LI-high and/or p53-positive tumors). The disease-free survival was significantly longer in the favorable-phenotype group than in the unfavorable-phenotype group (HR, 8.8; 95% CI, 1.9–40.4; *P* = 0.0007).

Figures 3A and 3B show the DFS and OS curves for the 4 combined Ki67-p53 status groups. Patients with Ki67 LI-low and p53-negative tumors survived longer than those in the other 3 groups (the patients with Ki67 LI-low and p53-positive tumors, Ki67-high and p53-negative tumors, and Ki67-high and p53-positive tumors) in both the DFS (*P* < 0.0001, *P* < 0.0001, and *P* = 0.0005, respectively; Figure 3A) and OS (*P* = 0.0010, *P* = 0.011, and *P* < 0.0001, respectively; Figure 3B) analyses. The DFS and OS curves therefore differed significantly between the patients with favorable-phenotype tumors and those with unfavorable-phenotype tumors (DFS: HR, 9.3; 95% CI, 3.5–24.5; log-rank *P* < 0.0001; Figure 3C, and OS: HR, 8.8; 95% CI, 1.9–40.4; log-rank *P* = 0.0007; Figure 3D). This difference was much more distinct than that between the low- and high-Ki67-LI curves (DFS: HR, 3.6; 95% CI, 1.7–7.9, and OS: HR, 4.4; 95% CI, 1.3–14.6). The 5- and 10-year OS rates were 97% and 97%, respectively, for the patients with favorable-phenotype tumors but only 91% and 65%, respectively, for the patients with unfavorable-phenotype tumors.

We next conducted subgroup analysis of HR-positive and HER2-negative breast cancer patients who had and had not received pre- or post-operative chemotherapy. Among the 49 patients who had received chemotherapy, those with favorable-phenotype tumors had significantly longer DFS than those with unfavorable-phenotype tumors (*P* < 0.0001). And then, among the 93 patients who had not received chemotherapy, those with favorable-phenotype tumors had significantly or almost significantly longer DFS than those with unfavorable-phenotype tumors (*P* = 0.0002).

Finally, we performed multivariate analyses of survival using Cox’s model of the proportional hazards regression including immunohistochemical parameters (combined Ki67-p53 status, FOXA1, GATA3, basal phenotype marker and P-cadherin) and the established clinicopathological factors (tumor size, lymph-node metastasis, nuclear grade and chemotherapy).

In those analyses, combined Ki67-p53 status, tumor size and chemotherapy was a significant prognostic indicators of DFS (*P* < 0.0001, *P* = 0.0001 and *P* = 0.0028, respectively; Table 3) and combined Ki67-p53 status was an only significant prognostic indicator of OS (*P* = 0.0081, Table 4).

**Table 3:** Univariate and multivariate analyses of disease-free survival in patients with hormone receptor-positive/HER2-negative primary breast cancer

			**Univariate**			**Multivariate**		
		**Total (n=142)**	**Hazard ratio**	**(95% CI)**	***P*-value**	**Hazard ratio**	**(95% CI)**	***P*-value**
Ki67-p53	Low Ki67 LI and Negative p53	88	1			1		
	High Ki67 LI and/or Positive p53	54	9.3	3.5-24.5	< 0.0001	11.6	4.2-32.3	< 0.0001
FOXA1	Low	20	1					
	High	119	1.4	0.51-3.6	0.54			
GATA3	Negative	30	1					
	Positive	112	1.5	0.65-3.5	0.34			
Basal phenotype marker	Negative	9	1					
(CK5/6, CK14, EGFR)	Positive	133	1.0	0.24-4.3	0.98			
P-cadherin	Low	90	1					
	High	52	1.3	0.59-2.8	0.52			
Tumor size	<5.0 cm	17	1			1		
	≧5.0 cm	122	4.9	2.1-11.4	0.0003	5.7	2.3-14.1	0.0002
Lymph-node metastasis	(−)	80	1					
	(+)	59	3.8	1.6-8.8	0.0020			
Nuclear grade	1, 2	111	1					
	3	31	4.2	1.9-9.0	0.0002			
Chemotherapy	No	93	1			1		
	Yes	49	2.6	1.2-5.7	0.014	3.5	1.5-7.9	0.0028

Abbreviation: *95%CI* 95% confidence interval, *LI* labeling index.

**Table 4:** Univariate and multivariate analyses of overall survival in patients with hormone receptor-positive/HER2-negative primary breast cancer

			**Univariate**			**Multivariate**		
		**Total**	**Hazard**	**(95% CI)**	***P*-value**	**Hazard**	**(95%CI)**	***P*-value**
		**(n=142)**	**ratio**			**ratio**		
Ki67-p53	Low Ki67 LI and Negative p53	88	1			1		
	High Ki67 LI and/or Positive p53	54	8.8	1.9-40.4	0.0049	7.9	1.7-36.7	0.0081
FOXA1	Low	20	1					
	High	119	0.49	0.06-3.9	0.50			
GATA3	Negative	30	1					
	Positive	112	1.1	0.31-4.3	0.84			
Basal phenotype marker	Negative	9	1					
(CK5/6, CK14, EGFR)	Positive	133	1.2	0.15-9.3	0.86			
P-cadherin	Low	90	1					
	High	52	1.8	0.57-5.5	0.32			
Tumor size	<5.0cm	17	1					
	≧5.0cm	122	3.4	0.90-12.8	0.071			
Lymph-node metastasis	(−)	80	1					
	(+)	59	3.9	1.02-14.7	0.045			
Nuclear grade	1, 2	111	1					
	3	31	3.8	1.2-11.9	0.0020			
Chemotherapy	No	93	1					
	Yes	49	2.0	0.65-6.3	0.22			

Abbreviation: 9*5%CI* 95% confidence interval, *LI* labeling index.

## Discussion

The international expert panel at the 2009 St. Gallen Consensus meeting referred to the importance of proliferation markers in deciding whether to include adjuvant chemotherapy in the treatment of patients with HR-positive HER-2-negative breast cancers. Several large-scale studies have evaluated the clinical significance of the Ki67 LI among patients with HR-positive breast cancer [7,18,21]. In this study, we showed that an IHC panel comprising p53 status and Ki67 LI is more accurate than Ki67 LI alone at predicting the prognosis for patients with HR-positive and HER2-negative breast cancer.

The results of our IHC panel divided the patients with HR-positive and HER2-negative invasive breast cancers into 2 distinct prognostic subtypes, those with favorable-phenotype (Ki67 LI-low and p53-negative) and unfavorable-phenotype group (Ki67 LI-high and/or p53-positive) tumors. Multivariate analysis showed that the IHC panel results, tumor size and chemotherapy were independent prognostic factors for DFS and that the IHC panel results was an only independent prognostic factor for OS. Furthermore, 76 of the 78 patients (97%) with early-clinical-stage (I or II) cancers showing the favorable phenotype were alive at the end of this study. The results of a similar immunohistochemical biomarker panel for 6 markers, including p53 and Ki67, were reported by Brian et al. to be a significant prognostic factor [32]. Ross et al. also showed that the immunohistochemical detection of 5 markers, including p53, was significantly associated with clinical outcome [33]. These reports support our data, at least in part; moreover, our immunohistochemical panel using 2 easy-to-use antibodies (Ki67 and anti-p53 antibodies) was both simpler than the cited panels. Miller et al. reported the similar results to this reports [24]. They evaluated the three molecular marker (Ki67, p53 and HER2) using the whole cases with HR-positive tumors. On the other hand, we excluded the HER2-positive tumors from the whole HR-positive tumors and then evaluate the clinicopathological implication of combined Ki67-p53 status in the patients with HR-positive and HER2-negative tumors. Nowadays, the patients with HER2-positive tumors are treated with anti-HER2 drugs and show different clinical outcome to those with HER2-negative tumors. So, our results give the more precise information and are more applicable to the dairy practice than the results of Miller et al.

The results of the immunohistochemical panel divided the HR-positive and HER2-negative breast cancer patients as follows: the 10-year DFS rates were 81% for the favorable phenotype group and 46% for the unfavorable phenotype group, while the 10-year OS rates were 97% and 65% for the favorable and unfavorable phenotype groups, respectively. To exclude the influence of adjuvant chemotherapy on the predictive value of the panel, we examined the prognostic significance of the panel separately in patients who received either pre- or post- operative chemotherapy and those who received no chemotherapy. As the immunohistochemical panel results were also identified as a significant prognostic factor in the patients who did not receive chemotherapy, we were able to exclude the influence of chemotherapy on our results. Our data indicate that patients with favorable-phenotype cancers have a clinical choice to avoid cytotoxic chemotherapy, as the baseline prognosis with adjuvant hormonal therapy alone is very good for this group.

In this report, the unfavorable phenotype-tumors exhibited significantly higher rates of positivity for HER2, basal phenotype markers (CK5/6, CK14, and EGFR), and P-cadherin than did the favorable-phenotype tumors. P-cadherin has been previously shown to be overexpressed on basal-type tumors [14,34]. These properties suggest that unfavorable-phenotype tumors take on not only the “HER2” phenotype [7] but also the “basal” phenotype. To our knowledge, this is the first clinical study to reveal an obvious correlation between luminal subtype-B and basal-type breast tumors. Basal-type tumors exhibit more *p53* mutations [9,35] and nuclear p53 protein accumulation [36] than do luminal-type or HER2-type tumors, so *p53* mutation is thought to be one of the characteristics of basal-type tumors [37,38]. Our results would be consistent with this viewpoint. We suspect that some tumors should be considered “mixed intrinsic subtype” tumors, that is, tumors that exhibit characteristics of 2 or more intrinsic subtypes and therefore cannot be classified as any “pure” intrinsic subtype.

## Conclusions

In conclusion, our results revealed that the cases with Ki67 LI-high and p53 positive showed a mixed tendency towards the “HER2” and “basal” types ,and that a simple immunohistochemical panel comprising Ki67 and p53 could distinguish between the cases with a favorable phenotype group and those with an unfavorable phenotype group among HR-positive and HER2-negative breast cancer patients. These suggest that our simple immunohistochemical panel comprising Ki67 and p53 is a promising tool for distinguishing between “luminal-subtype-A” and “luminal-subtype-B” breast cancers and management of patients with HR-positive breast cancer.

## Abbreviations

Ki67 LI: Ki67 labeling index; IHC panel: Immunohistochemical panel; HR: Hormone receptor; ER: Estrogen receptor; PgR: Progesterone receptor; TMA: Tissue microarray.

## Competing interests

The authors have declared no conflicts of interest.

## Authors’ contributions

TK and KI conceived of the study, performed experiments, analyzed data and wrote the manuscript. TM, TY and JY provided samples, collected clinical and pathological data. HT participated in designing the study and revising the manuscript. OM participated in the overall design, study coordination and finalized the draft of the manuscript. All authors read and approved the final manuscript.

## Pre-publication history

The pre-publication history for this paper can be accessed here:

http://www.biomedcentral.com/1472-6890/13/5/prepub
